# Dispensatorium Pharmaceuticum Brunsvicense, jussu principis in normam præscriptum atque ordinatum

**Published:** 1781-03

**Authors:** 


IV. *Diſpenſatorium Pharmaceuticum Brunſvicenſe, juſſu principis in normam præſcriptum atque ordinatum.* Brunſvici 1777, 4to. 378 pages.

THE chief merits of the work before us ſeem to be its containing an account of many new compoſitions, and its being written with great accuracy.

We learn from the preface that the authors have omitted ſeveral compoſitions becauſe they are now out of faſhion; this is certainly a very inſufficient reaſon, it being well-known that nothing is more variable than the faſhion and modes of medical practice. They have retained others, the efficacy of which is doubtful, or at leaſt not generally acknowledged, ſuch for inſtance are thoſe introduced of late years by Baron Störck.

In this compilation the authors have made uſe of other diſpenſatories, particularly thoſe of Wurtemberg, Augſburg, Paris, &c. but there are ſome of greater merit than theſe, as the Pharmacopœia copœia Suecica, for inſtance, of which no notice ſeems to have been taken.

The work conſiſts of two parts; the firſt is employed on the *materia pharmaceutica*, and extends through 168 pages; the ſecond, containing the compound medicines, occupies the remainder of the volume. The former of theſe is compiled with great induſtry. To the officinal name of each article, the authors have every where added not only the ſyſtematic name employed by Linnæus, but likewiſe the German and French names, and the ſynonyma of different authors. They are likewiſe careful to point out the compoſitions of which each ſimple is an ingredient.

On looking over the catalogue of ſimples, we are pleaſed at meeting with the quaſſia, columbo, and Lopez root; but on the other hand we obſerve a variety of others, ſuch as the lithoſpermum, monarda, colubrino, digitalis, &c. which we think had better have been omitted.

Under the head *helleborus niger* we find a very curious and intereſting remark, that ſerves to point out the difference of this ſpecies from the *Adonis vernalis* of Linnæus, or Hellebore of Hippocrates, which it ſeems is frequently ſubſtituted in its ſtead. The characters of both plants are given as follows: " *Radix veri Hellebori nigri*

capite

" capite nigro, ſtriato, avellanæ nucis magnitu-

" dine, plurimiſque quodamodo lævibus nigriſ-

" que fibris conſtat; levis ac rarioris texturæ,

" odoris fortis, ſaporiſque amaricantis, nauſeoſi.

" *Hellebori* vero ſic dicti *Hippocatris* (*Adonis Ver-*

" *nalis* Linn.) *radix*, quæ Helleboro nigro vero

" frequenter ſubſtituitur, habet caput exiguum,

" fibraſque craſſiores et copioſiores, coloris exte-

" rius minus atri, et interius minus albi; ſed po-

" tius griſei, aut ex albo flaveſcentis; ſubſtantiæ

" vero longe tenacioris." This diſtinction may ſerve to teach us how careful phyſicians ought to be in making experiments with plants recommended by others, and how neceſſary it is that every one who recommends a new plant to the trial of practitioners, ſhould deſcribe it with accuracy. The authors recommend the root of the *atropa mandragora* Linn. as an excellent remedy for indurated glands, when uſed in the form of a poultice.

The ſecond part of the work contains an immenſe number of good and bad formulæ, collected for the moſt part from different diſpenſatories. Among the good ones we may diſtinguiſh the *aqua ſtettinenſis ad uſus externos*; the *balſamum ſchauerianum*, kept formerly as a ſecret, but now purchaſed of the proprietor and publiſhed lished; *decoctum aperiens*, prepared from equal parts of rhubarb and madder root; *elixir vitrioli Halleri*; *decoctum ad fungos articulorum Heisteri*; *decoctum strobulorum pini Hirscheli*; *infusum corticis Peruviani frigidum.*

Among the extracts we find those of *cicuta*, *hyoscyamus*, *aconitum*, *belladona*, and *nux juglans immatura*, prepared simply from their expressed and inspissated juices. Those of *arnica*, *quassia*, *valeriana*, &c. are directed to be made by boiling the plants in water.

In their directions for preparing the *liquor vini probatorius*, the authors very properly observe, that it loses its properties when kept too long, and they caution the operator not to prepare it in a close room, on account of the suffocating smell that attends the process.

Under the title of *pilulæ purificantes*, we meet with the following formula: ℞ pulv. alterant. Edinburg. extr. fumariæ ā ā ℥ ji. extr. centaur. min. g. guaiac. resin. guaiac. terebinth. coct. ā ā ʒ ij. M. The authors give a full and exact description of an excellent soap for internal use, which we could wish to see introduced into the shops, instead of that commonly employed.

The

The following is their formula for preparing *Eau de luce*, to which they give the name of *ſpiritus ſalis ammoniaci ſuccinnatus.* "℞. Salis "tartari ʒiij. ol. ſuccini veri ʒiſs. terantur in "mortar. vitreo, guttatim ſucceſſive inſtillando "ſpiritus vini rectificati uncias quatuor, infundan-"tur in lagenam vitream, leviter obturatam. Di-"gerantur per quadrantem horæ, ſuper cineribus "calidis. poſtea liquor ſupernatans provide de-"cantetur et ſervetur. ℞. Spiritus ſalis ammo-"niaci cum calce viva parati ℥iſs; inſtillentur "liquoris antea parati guttæ ſexagint. M."

The *ſyrupus balſamicus*, prepared according to Hoffmann's preſcription, from julep of roſes, and the eſſence of balſam of Peru, we cannot but approve, on account of its ſimplicity. After this follow a variety of ſyrups, tinctures, and ointments: we ſhall here tranſcribe one of the latter, the *unguentum ad cancrum exulceratum Norfordii*, becauſe in a diſeaſe, ſo dreadful as the cancer is, and for which we know of no remedy, every thing ſeems to merit a trial; "℞. Succi ſe-"minis ricini, recens expreſſi, libram unam; ex-"ponatur in phiala plumbea radiis ſolaribus "tamdiu, donec olei conſiſtentiam acquirat. "Tunc immiſceantur, ſucci hujus inſpiſſati, "unciæ uni plumbi uſti, mercurii albi cum

" aqua calcis vivæ præcipitati, ā ā ſcrupulnm

" unum. M."

Towards the end of the volume we find a uſeful catalogue of officinals, with the ſyſtematic, German, and French names of each; and the work cloſes with a table of the prices at which the Brunſwick apothecaries are obliged to vend their medicines.

Upon the whole, this publication may be ſaid to contain many intereſting articles, and may prove very uſeful to any future compilers of a ſimilar work.

